# Watering-Place Wisdom, for Next Summer’s Practice

**Published:** 1872-10

**Authors:** 


### WATERING-PLACE WISDOM, FOR NEXT SUMMER'S PRACTICE.

THE beautiful October finds almost all home again; a home valued more than when it was left, for its tidiness, its quiet comfort, its freedom, and its unrestraint. You are sure now that everything not only looks, but is clean. You are certain, too, of clean sheets, of unbugged beds, and undisturbed sleep. No more tinkling of bells and banging of doors, late and early; no heavy tramps along the corridors after the midnight train; no unearthly screechings of locomotives; no heavy thumps of tables, and chairs, and ponderous boots, overhead, to rouse you from your first sleep; no breaking in upon the delicious dreaminess of the early morning, by the hasty, remorseless door-taps of the porter, and the quick footsteps of the

DAYLIGHT DEPARTERS.

No longer is it necessary to remain in full dress "from morn till dewy eve," for the old time abandon is admissible once more.

No interminable waitings for the cooking of what has been ordered; no more servants to fee every other day, to prevent unendurable annoyances and imminent starvation; no longer any dread, in dressing, of tearing the garment into ribbons for want of room to step or turn around, without deliberately surveying the situation and determining the space between trunk and toilet-table. You can sit down, or stand up, or cross the floor, without being scrutinized from top to toe, or having an eyeglass leveled at you, with the consciousness that it is for the purpose of determining your status in

PERSON, PROPRIETY, PURSE, AND POSITION.

But as others are doing to you what you instinctively and very promptly do to them, you let that pass as a universal right in this "land of the free and home of the brave," and, for that matter, in all other lands. On these and many other accounts, you really feel that home is simply delicious; in fact, you almost come to the conclusion that you will never leave it again, and in that conclusion you will unalterably remain until the first of June, when you will begin to turn your eye about for some place to get away from household care, and the worry of servants, who, taken all in all, are just about as perfect as yourself, no more, no less, considering the difference of opportunities, and culture, and learning. This you won't believe, however, but we will not argue the case just now, for we have not space, having wanted to tell you from the first a very unwelcome truth, that you are going to get sick, probably within a week; you and your children, too, although you are so much stronger and better than when you left home; because the excitement of travel, of constantly seeing new things, of having new food, and new modes of preparation almost every day, of being out in the open air from morning to night, and more or less on your feet and in motion during part of every hour in the day, every now and then being compelled to make unwilling efforts in getting seats, making connections, looking after the baggage and other things, all have conspired to give you a tremendous appetite, and for a few days or a week you will indulge it three times a day, although you are not out of doors one third of the time, and do not take one third of the muscular exercise to work off what you eat. Hence, as an inevitable result, you get fuller and fuller every day, the blood more and more impure, until you are absolutely sick, notwithstanding when you got home, you felt so well; and then you half conclude that it don't pay to spend so much time and money, and have so much bother, for so short a duration of health. But you commit a great error. You have laid in a stock of vigor and of re-creation which ought to last you until next year, and would last you, with a little judiciousness in the matter of eating, thus wise, at least until cold weather sets in, say about a week before Christmas, and from that time you can better afford to eat anything and everything: From the day you get home from your summer excursion, require every member of the household to eat at three regular hours every day, at five or more hours' interval; not a morsel of food of any kind between; no desserts; and at the last meal of the day, for adults, one cup of warm drink and an ordinary slice of cold bread and butter, or a ship biscuit, broken into the drink, and nothing else whatever. This will insure, in almost all cases, a night of good sleep, a vigorous appetite for breakfast, and a reasonable one for dinner, some six hours later. Such a course will result in a vigorous digestion, pure blood, and good health.

